# Effects of *Flos carthami* on CYP2D6 and on the Pharmacokinetics of Metoprolol in Rats

**DOI:** 10.1155/2011/207076

**Published:** 2010-11-28

**Authors:** Gaofeng Liu, Yan Liu, Rui Liu, Feng Dong, Zhiren Zhang

**Affiliations:** Department of Pharmacy, The Second Affiliated Hospital, Harbin Medical University, Harbin 150086, China

## Abstract

*Flos carthami* is a traditional Chinese herbal medicine. Clinically, the *Flos carthami* Injection has been used concomitantly with other Western drugs and may be used concomitantly with *β*-blockers, such as metoprolol, to treat cerebrovascular and coronary heart diseases, in China. Metoprolol is a CYP2D6 substrate and is predominantly metabolized by this isozyme. However, we do not know whether there is an effect of *Flos carthami* on CYP2D6 and the consequences of such an effect. Concern is raised regarding the possible herb-drug interaction. In this report, the effects of *Flos carthami* on the activity of CYP2D6 *in vivo* and *in vitro* and on the pharmacokinetics of metoprolol, in rats, are investigated. To assess the inhibitory potency of *Flos carthami*, the concentration associated with 50% inhibition (IC_50_) of dextromethorphan metabolism was determined based on the concentration-inhibition curves. The inhibitory effect of *Flos carthami* on CYP2D6 was also compared with cimetidine *in vitro*. *Flos carthami* could significantly inhibit CYP2D6 in rats both *in vitro* and *in vivo* (*P* < .05) and could slow down the metabolic rate of metoprolol as suggested by prolonged t_1/2_ (67.45%), by increased *C*_max_ (74.51%) and AUC_0−∞_ (76.89%). These results suggest that CYP2D6 is a risk factor when *Flos carthami* is administered concomitantly with metoprolol or other CYP2D6 substrates.

## 1. Introduction

Traditional herbal medications have been used concomitantly with Western drugs to treat a variety of diseases in China and other countries [1–4]. One of the concerns of healthcare professionals is that there is a lack of knowledge with regard to the pharmacokinetics of herbs, and there is a high possibility of herb-drug interaction [5]. This herb-drug interaction may significantly alter the efficacy and safety of the medications and result in therapeutic failure or toxicity [6–9]. Unfortunately, the availability of such information is very limited. It is well known that cytochrome P450 (CYP450) enzymes play a major role in drug-drug interactions. CYP2D6 isoform is one of the important members of the CYP450 family and mediates metabolism in almost 25% of the CYP450-metabolized drugs [10]. To date, CYP2D6-mediated drug-drug interactions have been extensively investigated [11]. However, there are only a few published CYP2D6-mediated studies with regard to the herb-drug interactions [12–15].

*Flos carthami* Injection is prepared from *Flos carthami*, a traditional Chinese herbal medicine. Clinically, *Flos carthami* Injection has been used in combination with *β*-blockers, such as metoprolol, to treat cerebrovascular and coronary heart diseases in China. Metoprolol is a CYP2D6 substrate and is predominantly metabolized by this isozyme. However, we do not know whether there is an effect of *Flos carthami* on CYP2D6 and the consequences of such an effect on its substrates. To this end, we have investigated the effects of *Flos carthami* on CYP2D6 and on the pharmacokinetics of metoprolol in rats. Our results show that *Flos carthami* exhibits an inhibitory effect on CYP2D6 and could slow down the metabolic rate of metoprolol.

## 2. Methods

### 2.1. Chemicals and Reagents

*Flos carthami* Injection (each milliliter injection contained 0.5 g *Flos carthami*) was purchased from Shanxi Yabao Pharmaceuticals Company (Shanxi, China). Dextromethorphan, dextrophan, *β*-glucuronidase, isocitric dehydrogenase, and DL-isocitric acid trisodium were obtained from Sigma Chemical Co. (St. Louis, Mo, USA). NADPNa_2_ was purchased from Geneview (Texas, USA). Metoprolol tartrate tablets were purchased from AstraZeneca Pharmaceuticals Company (Jiangsu, China). Metoprolol tartrate standard, bisoprolol fumarate standard, and buprenorphine standard were obtained from the National Institute for the Control of Pharmaceutical and Biological Products (Beijing, China). All other reagents were of analytical or HPLC grade.

### 2.2. Animals and Treatments

Wistar rats (180 ± 20 g, male) were supplied by the Animal Experimental Center of the Harbin Medical University, which was fully accredited by the Institutional Animal Care and Use Committee (IACUC) and handled in a manner that met all the recommendations formulated by the National Society for Medical Research and Guidelines for the Care and Use of Laboratory Animals. The rats were randomly divided into two groups (*Flos carthami*-treated and blank control) with eight rats in each group. Treated and control groups were administered a 1.8 mL/kg *Flos carthami* Injection and physiological saline by caudal vein for seven days, respectively. On day eight, the animals in both groups were given dextromethorphan (6 mg/kg) orally. Eight hours later, the rats were anesthetized with 4% halothane, and the chest was opened for removal of the liver. (the rats were fasted for 24 h before being killed.) The urine and liver of each rat were collected and stored at −20°C or −80°C for further analysis.

### 2.3. Sample Treatments and HPLC Conditions

One milliliter of urine with 50 *μ*L internal standard buprenorphine (2 mM) was mixed with 1 mL sodium acetate buffer (pH 5.0) and incubated at 37°C overnight. After incubation, the urine was mixed with 1 mL NaOH (3 M), extracted with 3 mL extraction mixture (n-hexane: n-butanol, 9 : 1, v/v), and centrifuged for 5 min at 3000 × g. Two hundred microliters of HCl (0.01 M) was added to the organic phase, and the mixture was centrifuged for 5 min at 3000 × g. The aqueous phase of 20 *μ*L was injected into the Waters HPLC system 2010 (Waters, USA) with a fluorescence detector, at an excitation wavelength of 280 nm and an emission wavelength of 310 nm. Dextromethorphan, dextrophan, and buprenorphine were separated on a Hypersil-C_6_H_5_ column (4.6 mm × 250 mm, 5 *μ*m, Elite Co.). The mobile phase, at a flow rate of 1 mL/min, consisted of 0.02 M potassium dihydrogen phosphate, 0.02 M sodium hexanesulfonate, acetonitrile, and methanol (33 : 33 : 100 : 94, v/v/v/v).

### 2.4. Microsomal Preparation and Incubation Conditions

Microsomes were prepared by differential centrifugation as described previously [16]. The protein content was quantified according to the method of Bradford [17]. Liver microsomes were resuspended in an assay buffer (20 mM DL-isocitric acid trisodium, 1 × 10^3^ U/l isocitric dehydrogenase, and 1 mM MgCl_2_) and incubated at a final protein concentration of 1 mg/mL (500 *μ*L final volume) for 45 min at 37°C, with 0.3 mM dextromethorphan and 1 mM NADP^+^. The reactions were terminated by acidification with ice cold 20% trichloroacetic acid. After incubation, 100 *μ*L liver microsomes solution was mixed with 50 *μ*L internal standard buprenorphine (2 mM) and 100 *μ*L NaOH (3 M) extracted with 2 mL extraction mixture (n-hexane: n-butanol, 9 : 1, v/v) and centrifuged for 5 min at 3000 × g. One hundred and fifteen microliters of HCl (0.01 M) were added to the organic phase, and the mixture was centrifuged for 5 min at 3000 × g. The aqueous phase of 20 *μ*L was injected into the Waters HPLC system 2010 for analysis. The HPLC conditions were the same as described earlier (see Section 2.3).

### 2.5. Inhibition Curve Study

Dextromethorphan was incubated with *Flos carthami* Injection at concentrations of 0, 5, 10, 15, 20, 25, 30, and 45 mg/mL under the conditions described earlier, with triplicate incubations for each concentration. After incubation and sample treatment, a 20 *μ*L sample was injected into the Waters HPLC system 2010 for analysis. The plot was the logarithm of *Flos carthami* Injection concentration (*X*) versus the inhibition ratio of *Flos carthami* on CYP2D6 (*Y*). To assess the inhibitory potency of *Flos carthami*, the concentration associated with 50% inhibition (IC_50_) of dextromethorphan metabolism was determined based on the concentration-inhibition curve.

### 2.6. Comparison of the Inhibitory Activities of *Flos carthami* with Cimetidine

The liver microsomes were divided into three groups: blank group, *Flos carthami*-treated group (*Flos carthami *Injection was added to the blank liver microsomes; final concentration was 30 mg/mL), and the cimetidine-treated group (cimetidine was added to the blank liver microsomes; final concentration was 0.6 mg/mL). The incubation conditions were the same as described earlier (see Section 2.4), and the metabolic rates of dextromethorphan were compared.

### 2.7. Effects of *Flos carthami* on Pharmacokinetics of Metoprolol

A total of 16 rats were randomized into two groups, *Flos carthami *treated and blank control. In *Flos carthami* Injection, 1.8 mL/kg was administered by the caudal vein once daily for seven days. One milliliter of physiological saline was given in the blank control group. On day eight, metoprolol 25 mg/kg was administered by gavage to both groups. The blood samples for the determination of plasma concentration of metoprolol were collected by caudal vein at 0 (predose), 0.25, 0.5, 1, 1.5, 2, 2.5, 3, 4, 6, and 9 hours after the dose of metoprolol and were put into heparinized test tubes. The plasmas were obtained by centrifugation of the blood samples and preserved at −20°C for further analysis. Bisoprolol fumarate standard (internal standard) solution (100 *μ*g/mL, 20 *μ*L), NaOH solution (2 mol/l, 200 *μ*L), and ethyl acetate (2 mL) were added to fresh blood plasma (300 *μ*L). Each sample was mixed thoroughly by vortex for 2 min and centrifuged for 10 min at 3000 rpm. Supernatant of 1.5 mL was taken out, and then 2 mL ethyl acetate was added again to the rest of the mixture. Each sample was again mixed thoroughly by vortex for 2 min, and the mixture was then centrifuged for 10 min at 3000 rpm, and a supernatant of 1.5 mL was taken out. The two supernatants were mixed, and sulfuric acid (1%, 100 *μ*L) was added to them. They were then mixed thoroughly by vortex mixing for 2 min and centrifuged for 10 min at 3000 rpm. The lower aqueous phase of 20 *μ*L was analyzed by HPLC. The HPLC conditions were Waters HPLC system 2010 (Waters, USA) with ultraviolet detector, at wavelength of 223 nm. Metoprolol and bisoprolol (internal standard) were separated on a Kromasil C_18_ column (4.6 mm × 250 mm, 5 *μ*m, Institute of Physical Chemistry in Dalian, China). The mobile phase, at a flow rate of 0.8 mL/min, consisted of methanol, 0.02 mol/l potassium dihydrogen phosphate, and 0.01 mol/l dipotassium hydrogen phosphate (56 : 22 : 22, v/v/v).

### 2.8. Statistical Analysis

The linear regression of experimental data *in vivo* and *in vitro* was performed according to the canonical correlation analysis (CCA). Data were expressed as means ± SD and analyzed by Dunnett's test. The metoprolol pharmacokinetics (PK) parameters were derived with a nonlinear regression iterative program, DAS 1.0 (Drug Clinical Evaluation Center, Anhui, China) pharmacokinetic statistical software. The derived PK parameters among the groups were statistically analyzed by ANOVA with Dunnett's test. *P* < .05 and *P* < .01 were considered to be statistically significant and very significant, respectively.

## 3. Results

### 3.1. In Vivo and In Vitro Study of Dextromethorphan Metabolism

**Figure 1:**
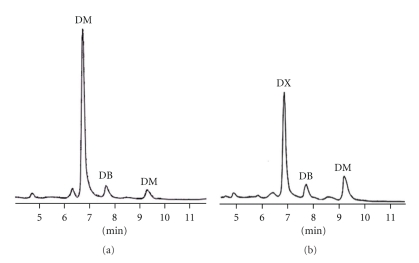
HPLC chromatograms of urine of rats *in vivo.* (a) Blank group, (b)* Flos carthami*-treated group. DX: dextrophan (metabolite of dextromethorphan); DB: buprenorphine (internal standard); DM: dextromethorphan.

**Figure 2:**
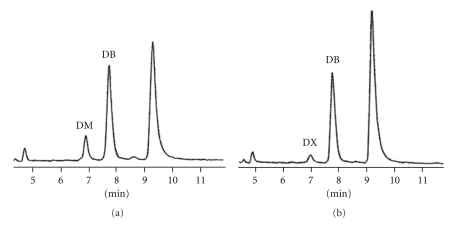
HPLC chromatograms of liver microsome of rats *in vitro. *(a) Blank group and (b) *Flos carthami*-treated group. DX: dextrophan (metabolite of dextromethorphan); DB: buprenorphine (internal standard); DM: dextromethorphan.

**Figure 3:**
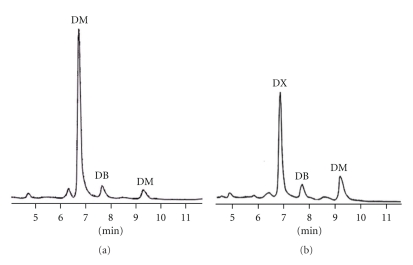
Linear regression of experimental data* in vivo* and *in vitro. *

Dextromethorphan, dextrophan, and buprenorphine in the urine and in the rat liver microsomes of blank control and the *Flos carthami*-treated groups were analyzed by HPLC. The chromatographies of dextrophan, dextromethorphan, and buprenorphine *in vivo* and *in vitro* of blank control and *Flos carthami*-treated rats were separated well (Figures 1 and 2). The rate of dextromethorphan metabolism in the treated group was lower than that in the control group both *in vivo* and *in vitro *(111.23 ± 26.20 in the *Flos carthami*-treated group and 160.95 ± 37.23 in the control *in vivo*; *n* = 8, *P* < .05; 0.037 ± 0.008 in microsomes prepared from the *Flos carthami*-treated rats and 0.068 ± 0.020 in control rats *in vitro*; *n* = 8, *P* < .05). The data for dextromethorphan metabolism *in vivo* and *in vitro *were further plotted as *X* and *Y *(*X*: dextrophan/dextromethorphan *in vivo; Y*: dextrophan/dextromethorphan *in vitro*) to reveal the correlation between the *in vivo* and *in vitro* findings. The results showed that the rate of dextromethorphan metabolism inhibited by *Flos carthami* correlated well *in vivo* and *in vitro *(*r* = 0.9389) (Figure 3). All data suggested that *Flos carthami* inhibited the activity of CYP2D6. 

### 3.2. Inhibition Curve and IC_50_ of *Flos carthami*

**Figure 4:**
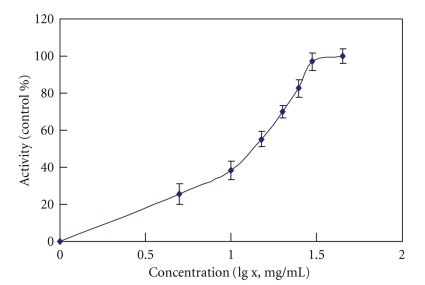
The inhibition curve of *Flos carthami *on dextromethorphan metabolic rate (mean ± SD, *n* = 8). The concentration of *Flos carthami* was ranging from 0, 5, 10, 15, 20, 25, 30, to 45 mg/mL, respectively.

The influence level of *Flos carthami* on dextromethorphan metabolism was investigated in rat liver microsomes prepared from *Flos carthami*-treated group and the blank control group. Dextromethorphan metabolism was inhibited in the *Flos carthami*-treated group compared to the control group in a dose-dependent manner. The IC_50_ value of *Flos carthami* was determined based on the concentration-inhibition curve (Figure 4) and was used to represent the inhibitory effect. The IC_50_ value was 10.64 mg/mL.

### 3.3. Comparison of Inhibitory Activities of Flos carthami with Cimetidine

**Figure 5:**
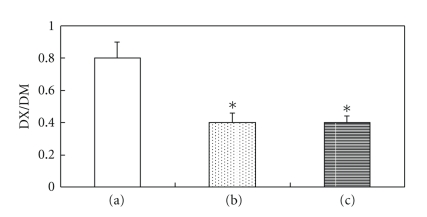
Comparison of the inhibitory activities of *Flos carthami* with cimetidine (mean ± SD; *n* = 8): (a) Blank group. (b) *Flos carthami*-treated group. (c) Cimetidine-treated group. *P < .05 versus blank group.

Our results showed that the dextromethorphan metabolic rate of the *Flos carthami*-treated group (30 mg/mL) was similar to that of the cimetidine-treated group (0.6 mg/mL). This result suggested that *Flos carthami* could inhibit CYP2D6 activity (Figure 5). 

### 3.4. Effects of *Flos carthami* on Pharmacokinetics of Metoprolol

**Figure 6:**
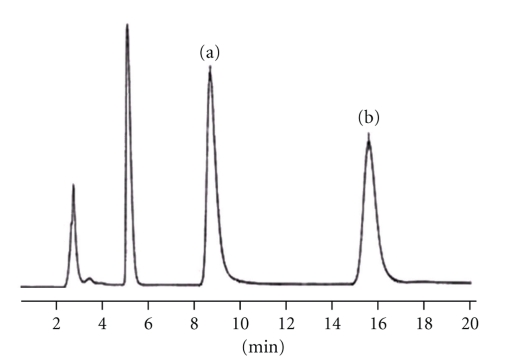
HPLC chromatogram of blood plasma of rats. (a) Metoprolol standard. (b) Bisoprolol standard.

**Figure 7:**
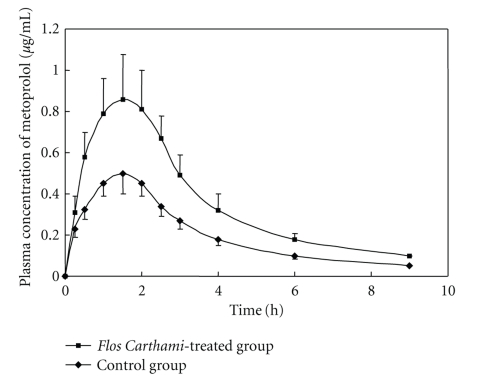
Mean plasma concentration-time profiles of metoprolol in control group and *Flos carthami*-treated group.

**Figure 8:**
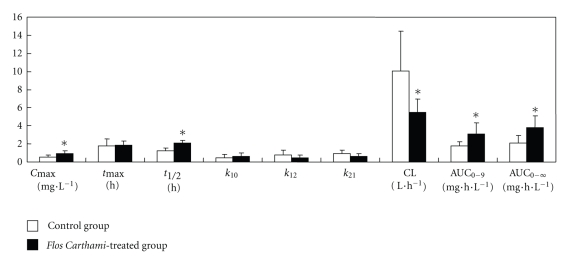
Pharmacokinetics parameters of metoprolol (mean ± SD; *n* = 8); *P < .05.

The chromatography of metoprolol and bisoprolol (internal standard) in blood plasma was shown in Figure 6. The mean metoprolol plasma concentration-time profiles were overlaid by the blank control group (without *Flos carthami* treatment) and the *Flos carthami*-treated group (Figure 7). The metoprolol mean plasma PK parameters were shown in Figure 8. 

## 4. Discussion

The present study addresses the fact that CYP2D6 is involved in the metabolism of *Flos carthami* in rats. One of the main findings of the study is that the rate of dextromethorphan metabolism in the *Flos carthami*-injected group is lower than that of the blank control both *in vivo* and *in vitro*. Dextromethorphan is widely used as a probe for CYP2D6 phenotyping and for the assessment of CYP2D6 activity [18]. All the data have implied that *Flos carthami *inhibits CYP2D6. 

For further investigation on the effect of *Flos carthami* on CYP2D6, inhibition curve experiments were performed using rat liver microsomes treated with the drug. The metabolism of dextromethorphan was inhibited in the rat liver microsomal reconstituted system treated with *Flos carthami*, in a dose-dependent manner. One of the most important findings of the current study is that *Flos carthami* inhibits the activity of CYP2D6.

Cimetidine is a specific CYP2D6 inhibitor [19]; hence, it was chosen to compare the inhibitory activities with *Flos carthami*, and the result showed that the dextromethorphan metabolic rate of 30 mg/mL* Flos carthami* was similar to that of 0.6 mg/mL cimetidine in the rat liver microsomal reconstituted system.

**Figure 9:**
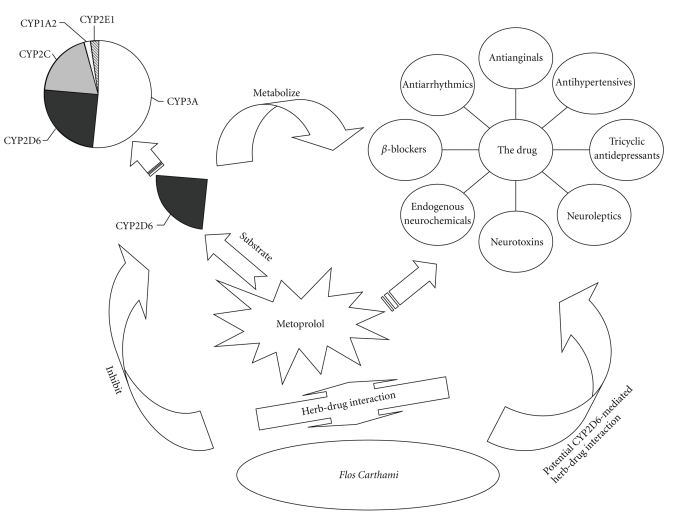
CYP2D6-mediated herb-drug interaction between *Flos carthami *and metoprolol or other CYP2D6 substrates.

*Flos carthami *(also called safflower) is a traditional Chinese herbal medicine and has extensive effects on the cardiovascular system, such as improving the microcirculation, anticoagulant, antihypertensive, antihyperlipidemia, antithrombotic, and hypoxia tolerance [20–23]. It is applicable in treatment of coronary heart disease, angina, myocardial infarction, hypertensive, atherosclerosis, cerebral thrombosis, and so forth [24]. Clinically, *Flos carthami* Injection is very common to be used concomitantly with other Western drugs and may be used in combination with *β*-blockers, such as metoprolol, to treat the cerebrovascular and coronary heart diseases in China [25]. Because metoprolol is a well-known CYP2D6 substrate, one has to consider the possible herb-drug interaction. In this paper, the effect of *Flos carthami* on metoprolol pharmacokinetics is investigated in rats. Our data show that* Flos carthami* slowed down the metabolic rate of metoprolol as suggested by prolonged t_1/2_ (67.45%), by increased *C*_max_ (74.51%) and AUC_0−∞_ (76.89%), in the *Flos carthami*-treated group as compared to the blank control. These results suggest that CYP2D6 is a risk factor when *Flos carthami* is administered concomitantly with metoprolol or other CYP2D6 substrates (Figure 9). The current study provides strong evidence that the inhibition of CYP2D6 by *Flos carthami *could result in elevated plasma concentration of coadministered drugs that are substrates of CYP2D6, which may cause adverse side effects due to the concentration of concomitantly used drugs over the limit of the toxicity threshold, especially for the drugs with narrow therapeutic windows.

CYP2D6 shows genetic polymorphism, and based on the CYP2D6 activity, human beings are categorized as ultrarapid metabolizers, extensive metabolizers, intermediate metabolizers, and poor metabolizers [26, 27]. CYP2D6 is clinically important in the metabolism of up to 25% of all clinical drugs, including *β*-blockers, antiarrhythmics, antianginals, antihypertensives, tricyclic antidepressants, neuroleptics, neurotoxins, and endogenous neurochemicals [11, 28, 29]. The inhibitory effect of* Flos carthami* on CYP2D6 activity could be associated with polymorphism feature of CYP2D6, which is clinically relevant when *Flos carthami* is administered with the drugs mentioned above or in individually tailored drug doses.

Besides its clinical application, *Flos carthami* has also been used as dietary supplements [23]. Therefore, it is important to note that *Flos carthami *from food sources may also alter the pharmacokinetics of other CYP2D6 substrates.

Several studies with regard to the effects of Kampo medications and botanical supplements on the CYP2D6 activity have been reported [30–32]. However, to the best of our knowledge, this is the first paper on a CYP2D6-mediated interaction between *Flos carthami *and Western drugs *in vivo*. Further investigation for the clinical significance is needed.
